# Hyperactivation of Akt/mTOR and deficiency in tuberin increased the oxidative DNA damage in kidney cancer patients with diabetes

**DOI:** 10.18632/oncotarget.1833

**Published:** 2014-03-17

**Authors:** Samy L. Habib, Sitai Liang

**Affiliations:** ^1^ Department of Geriatric, Geriatric Research, Education, and Clinical Center, South Texas Veterans Healthcare System; ^2^ Department of Cellular and Structural Biology, University of Texas Health Science Center at San Antonio San Antonio, TX

**Keywords:** Diabetes, RCC, Tuberin, mTOR, 8-OxodG and OGG1

## Abstract

Recent study from our laboratory showed that patients with diabetes are at a higher risk of developing kidney cancer. In the current study, we have explored one of the mechanisms by which diabetes accelerates tumorigenesis in the kidney. Kidney cancer tissue from patients with diabetes showed a higher activity of Akt and decreased in total protein of tuberin compared to kidney cancer patient without diabetes or diabetes alone. In addition, a significant increase in phospho-Akt/tuberin expression was associated with an increase in Ki67 expression and activation of mTOR in kidney tumor with or without diabetes compared to diabetes alone. In addition, decrease in tuberin expression resulted in a significant decrease in protein expression of OGG1 and increased in oxidative DNA damage, 8-oxodG in kidney tissues from patients with cancer or cancer+diabetes. Importantly, these data showed that the majority of the staining of Akt/tuberin/p70S6K phosphorylation was more prominently in the tubular cells. In addition, accumulation of oxidative DNA damage is localized only in the nucleus of tubular cells within the cortex region. These data suggest that Akt/tuberin/mTOR pathway plays an important role in the regulation DNA damage and repair pathways that may predispose diabetic kidneys to pathogenesis of renal cell carcinoma.

## INTRODUCTION

Epidemiological studies have shown that prior history of diabetes is associated with an increased risk of cancer [1-6]. Diabetes is also associated with a higher rate of mortality and cancer recurrence [7,8]. The retrospective International Renal Cell Cancer Study showed that a 5 to 10 year history of diabetes increased the relative risk for renal cancer by 40%, both in men and women [9]. Recent study showed that incidence and mortality rate of kidney cancer were increased by 47% and 43%, respectively, after age-adjusted [10]. A recent study from our laboratory showed that 25.4% of kidney cancer patients have diabetes (screening RCC patients from 1994-2009) indicting that diabetes is a major contributing factor in increasing the risk of kidney cancer [11].

Diabetes and hyperglycemia augment oxidative metabolism and free radical production. Increased oxidative stress in diabetes contributes to the pathogenesis of diabetic complications [12-15]. Reactive oxygen species (ROS) are potential upstream mediators of the effects of high glucose. ROS may introduce strand scission, deletions, insertions and rearrangements. ROS also induces mutations and can activate oncogenes, or inactivate tumor suppressor genes, altering the control of cell cycle events [16]. Several of the known ROS-induced base modifications are promutagenic [17,18]. Mutations are introduced via misincorporation of DNA bases, for example, due to the presence of unrepaired DNA adducts, or by slippage of DNA polymerase during replicative by-pass [19]. 8-OxodG is a major form of oxidative DNA that enhances lesions. 8-OxodG adducts formed by ROS primarily result in GC to TA transversions [20]. Several reports described an increase in 8-oxodG content in mononuclear cells and in urine in type I and type II diabetic patients [21,22]. Moreover, 8-oxodG levels are increased in kidneys of rats with type I diabetes [23,24]. OxodG is repaired by DNA repair enzyme, 8-oxoguanine-DNA glycosylase (OGG1). Previous study show that mutations in *OGG1* are associated with cancer and the efficacy of DNA repair may determine the susceptibility to carcinogens [25]. Our recent published data showed a significant association between mutations in OGG1 (Ser^326^ to Cys) and type II diabetes in genomic DNA isolated from blood mononuclear cells of diabetic patients. This suggests that OGG1 plays an important role in regulating the oxidative DNA damage in diabetic patients [26].

Tuberin (protein encodes by *TSC2*) expression was initially induced following acute renal injury, suggesting that the *TSC2* may also functions as an acute-phase response gene, limiting the proliferative response after injury [27]. Akt is directly phosphorylates/inactivates *TSC2* at Ser^924^, Thr^1462^ and Thr^1518^ [28]. Phosphorylation of *TSC2* by Akt disrupts the *TSC1-TSC2* complex and the subcellular localization of *TSC1* and *TSC2* as well as its function as tumor suppressor genes (28-30). Akt phosphorylated by PI-3K at Ser^473^ phosphorylates/inactivates *TSC2* and leads to the activation of mTOR [29]. The loss or inactivation of *TSC1/2* activates mTOR to phosphorylate its downstream kinase, p70S6K [31]. Recent study from our laboratory showed that induction of type I diabetes in rat and exposure of renal proximal tubular epithelial human cells to high glucose resulted in a decrease in the protein expression of DNA repair enzyme, OGG1 [32]. The mechanism by which diabetes enhances certain pathways to develop cancer is largely unknown.

## RESULTS

### Hyperactivation of Akt resulted in decrease total tuberin in kidney cancer patients with diabetes

**Fig 1:**
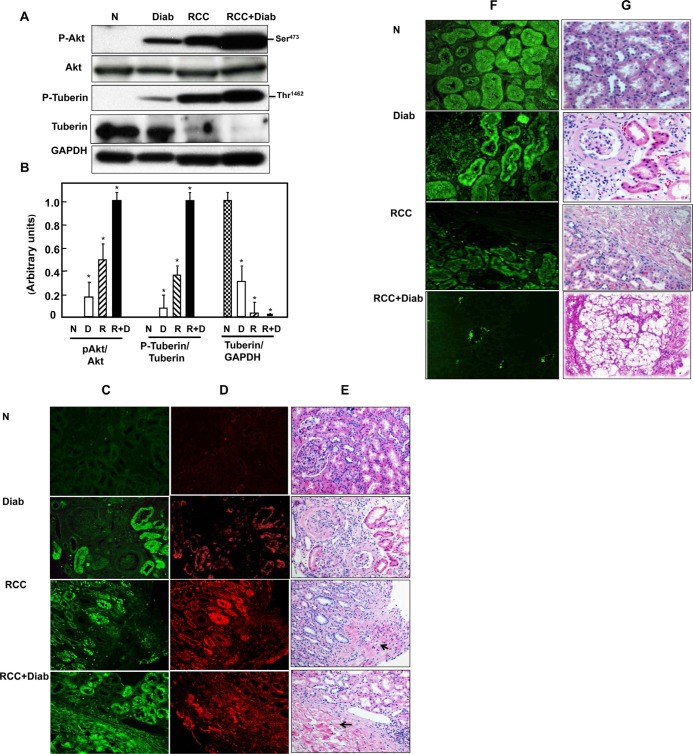
Hyperactivation of Akt resulted in decrease total tuberin in kidney cancer patients with diabetes (A) Representative Western blot analysis of kidney tissues from normal healthy subjects (N), diabetic (Diab), cancer (RCC) and cancer with diabetes (RCC+Diab). GAPDH was used as a loading control. (B) Histograms in the bottom panel showed the significant difference from normal healthy subjects (indicated by *P<0.01) to diabetic, cancer or cancer with diabetes. Kidney sections from normal control (C), diabetic (D), RCC and RCC+Diabetic patients were stained with phospho-Akt, phospho-tuberin and tuberin antibodies followed by FITC- anti-rabbit IgG as secondary antibodies. FITC green signals for phospho-Akt (C) and Alexa Fluor red signals for phospho-tuberin (D) were detected using a filter with excitation at 535 nm and 594 nm, respectively. Note that Akt/tuberin phosphorylation and tuberin staining is more prominent in the majority of the tubular cells. (F) Tuberin staining was less in tubular cells in different kidney sections of diabetic patient compared to the section from normal healthy subjects. In addition, tuberin staining was lost in kidney section from patients with RCC+diabetes compared slight staining in kidney section from patients with RCC alone. (E&G) H&E staining was performed in kidney sections (from matching kidney sections that was used for immunostaining) from healthy subjects, diabetic patients or kidney cancer patients with or without diabetes. Normal architecture of glomerular and tubular (distal and proximal cells) compartments was identified in kidney sections of normal (N) healthy subjects while nodular glomerulosclerosis, glomerular hypertrophy and tubular thickening in kidney section of diabetic patients (Diab). Kidney sections from cancer patients (RCC) were composed of clear cells with small nuclei and abundant cytoplasm set in a very prominent but delicate vasculature and homogeneous granular cells. Section from kidney cancer patients with diabetes (RCC+Diab) show clear cell are multinucleated with small round inform nuclei and tubules are filled with red blood cells marked with arrow.

Akt is major protein kinase that regulates a majority of cellular pathways including cell tumorigenesis. Akt plays a major role in both cell survival and resistance to tumor therapy. We have examined kidney tissue from normal (N) healthy people, patients with diabetes (Diab), patients with kidney cancer (RCC) without or with diabetes (RCC+Diab) for Akt activation/phosphorylation at Ser^473^. Data in Fig.1A showed that Akt was hyperactivated in kidney tissue from patients with kidney cancer and diabetes more than patients with kidney cancer alone. In addition, kidney cancer from patients with diabetes also showed hyperactivation of Akt in kidney cancer without diabetes and kidney from diabetic patients. Hyperactivation of Akt results in phosphorylation of tuberin at Thr^1462^. Hyper-phosphorylation of tuberin at Thr^1462^ showed the same trend as Akt phosphorylation in all kidney tissue from all four groups, indicating that Akt is the major kinase that phosphorylates and inactivates tuberin (Fig. 1A&B). In addition, phosphorylation of tuberin by Akt regulates tuberin-hamartin complexes and tuberin activity as a tumor suppressor protein lead to hamartin-tuberin degradation. Data in Fig. 1A&B shows that total tuberin was highly expressed in healthy subjects and significantly decreased in kidney tissue of diabetic patients while partially and significantly decreased in kidney cancer tissue without diabetes and in kidney cancer tissue with diabetes, respectively.

### Phosphorylation of Akt and tuberin are localized in tubular cells

Immunofluorescence staining was performed in all kidney sections from all four groups to identify which renal cells express higher levels of phospho-Akt and phospho-tuberin. A Higher staining of phospho-Akt (Fig. 1C) and phospho-tuberin (Fig. 1D) was detected in the distal and proximal tubular cells while no staining showed in glomerular compartments suggesting the cells' specificity. Sections from kidney cancer patients showed higher staining of phospho-Akt/tuberin mainly in adjacent tumor area than in the tumor areas (Fig. 1C&D) compare to kidney tissue from patients with kidney cancer or diabetes alone. Strong staining of phospho-proteins was detected in whole kidney section of cancer patients with diabetes confirming Western blot data (Fig. 1C&D).

### Histology of kidney section from diabetic and/or cancer patients

The histology of kidney sections from normal healthy subjects, diabetic patient or kidney cancer patient with or without diabetes was examined by H&E staining. Normal architecture of glomerular and tubular (distal and proximal cells) compartments is identified in kidney section of normal (N) healthy subjects while nodular glomerulosclerosis, glomerulus hypertrophy, and tubular thickening in kidney section of diabetic patients (Diab) (Fig. 1 E). Kidney sections from cancer patients were composed of clear cell type with small nuclei and abundant cytoplasm set in a very prominent but delicate vasculature and homogeneous granular cells (Fig. 1E). Sections from kidney cancer with diabetes (RCC+Diab) showed those clear cells are multinucleated with small round inform nuclei and tubules are filled with red blood cells (Fig. 1E).

Activated Akt phosphorylates tuberin at Thr1462 residues resulted in its dissociation from hamartin and its degradation [27]. A significant decrease in tuberin expression was detected in kidney tissue from diabetic patient compared to kidney tissue from normal healthy subjects (Fig.1A&B). These data was also confirmed by immunofluorescence staining of tuberin in kidney sections from all four groups. (Fig.1F). H&E staining was performed in kidney sections from all four groups show normal structure of kidney cells in normal healthy subject, nodular glomerulosclerosis, glomerular hypertrophy and tubular thickening in diabetic patients. Kidney tumor section showed a clear cell carcinoma and normal tubular cells (in adjacent tissue) cell type in patients without diabetes and tumor with clear cell type with prominent cell borders and prominent vasculature in kidney section of cancer patients with diabetes (Fig. 1G).

### Overexpression of ki67 protein in kidney cancer patients with diabetes

**Fig 2:**
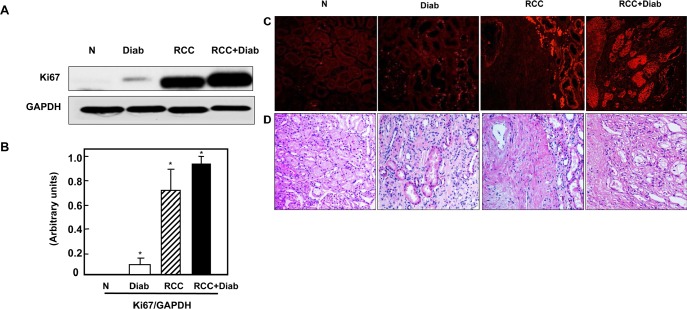
Overexpression of ki67 protein in kidney cancer patients with diabetes (A) Representative Western blot show higher expression of Ki67 protein expression in kidney tissue from cancer patients with or without diabetes while slight increase in kidney tissue of diabetic patients. (B) Histograms in the bottom panel show the significant difference from normal healthy kidney tissues to other groups is indicated by *P<0.01. (C) These data was confirmed by immunofluorescence staining of Ki67 in kidney paraffin sections from all four groups. Note that Ki67 staining is more prominent in the majority of the tubular cells. (D) H&E staining from the matching kidney sections of all four groups was performed to show the changes in histology of kidney sections from diabetic kidney tissue and tumor of cancer with or without diabetes.

The well-known cellular proliferation marker is Ki-67, which may exactly evaluate the proliferation activity of tumor cells. A significant increase in Ki67 expression was detected by Western blot analysis in kidney cancer tissue with or without diabetes while a slight increase was detected in kidney tissue of diabetic patient (Fig. 2A&B). These data was confirmed by immunofluorescence staining of Ki67 in kidney paraffin sections from all four groups. The majority of nuclear staining of Ki-67 was detected in tubular cells of diabetic kidney as well as in tubular cells of the adjacent tumor of kidney cancer with or without diabetes (Fig 2C). H&E staining from the matching kidney sections of all four groups was performed to show the changes in histology of kidney sections from diabetes and cancer with or without diabetes (Fig. 2D).

### Hyperactivation of mTOR in kidney cancer patients with diabetes

**Fig 3:**
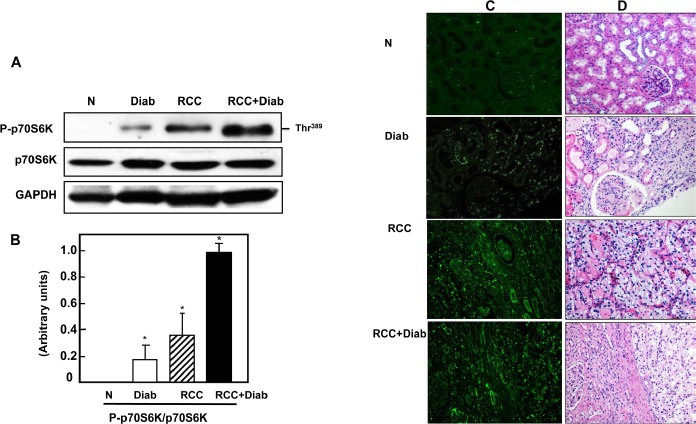
Hyperactivation of mTOR in kidney cancer patients with diabetes (A) Activation of mTOR as evidenced by the increased phosphorylation on Thr^389^ of p70S6K was analyzed by Western blot. Representative blots show hyperphosphorylation of mTOR (measured by phosphorylation levels of p70S6K at Thr^389^) was detected in kidney tissue from cancer patients with diabetes compare to a slight increase in diabetic patients and non-detectable expression in normal healthy subjects. (B) Histograms in the bottom panel show the significant difference from normal healthy kidney tissue to other groups of tissues is indicated by *P<0.01. (C) The increase in phospho-p706SK was confirmed by immunofluorescence staining which demonstrated, increased staining of phospho-p706SK in tubular cells of kidney sections from diabetic, kidney cancer with or without diabetes. (D) H&E staining from the matching kidney sections of all four groups was performed to show the changes in cell structure in diabetic kidney tissue and tumor histology of kidney cancer with or without diabetes.

mTOR signaling implicated in inflammatory, metabolic, degenerative, proliferative and cancer. Tuberin is an important signaling protein that is intimately involved in protein translation through regulating the mTOR pathway [13-15]. The induction of diabetes increased tuberin phosphorylation/inactivation and resulted in the activation of mTOR as evidenced by the increase phosphorylation of its downstream target p70S6K at Thr^389^. Slight increase in phosphorylation of p70S6K at Thr^389^ was detected in kidney tissue of diabetic patients compared to kidney tissue from normal healthy subjects (Fig. 3A&B). Hyper-phosphorylation of p706SK was detected in kidney tissue of cancer patients with diabetes compared to kidney tissue of cancer patients without diabetes (Fig. 3A&B). The increase in phospho-p706SK was confirmed by immunofluorescence staining which demonstrated by an increase of phospho-p706SK staining in kidney sections of patients with diabetes, cancer with or without diabetes (Fig. 3C). H&E staining from the matching kidney sections of all four groups was performed to show the changes in cell structure in diabetic kidney tissue and tumor histology of kidney cancer with or without diabetes (Fig. 3D).

### Significant accumulation of 8-oxodG and decreased in OGG1 protein in kidney cancer patients with diabetes

**Fig 4:**
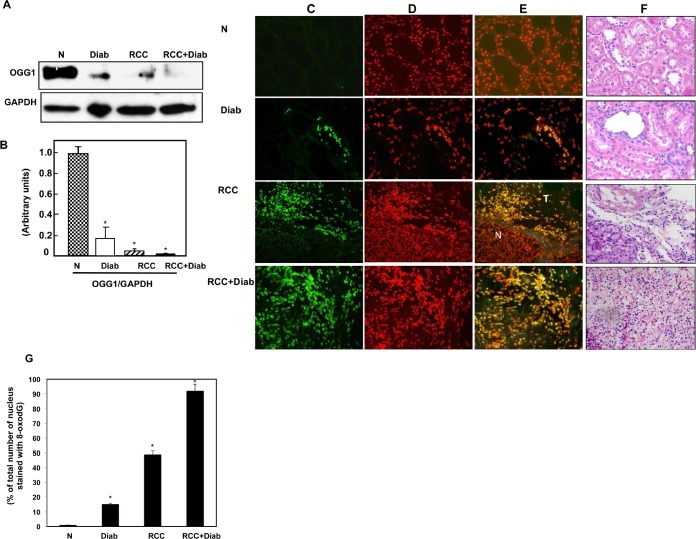
Significant accumulation of 8-oxodG and decreased in OGG1 protein in kidney cancer patients with diabetes (A) Protein expression of OGG1 was measured by Western blot analysis. The results showed a significant decrease in OGG1 expression in kidney tissue of cancer patients with diabetes while kidney tissue from cancer patients showed a slight expression. (B) Histogram showed the significant difference from normal healthy kidney tissue to other groups is indicated by *P<0.01. (C) Significant accumulation of 8-oxodG was detected in kidney sections of RCC+diabetes compared to RCC and diabetes alone and control healthy subjects. Kidney sections were double stained with 8-oxodG antibody followed by FITC-anti-rabbit IgG as secondary antibodies and with Propidium Iodide (PI) for nucleus. Note that 8-oxodG staining is more prominent in the majority of the tubular cells. (D) FITC (8-oxodG) green signals and PI red signals in nucleus were detected using a filter with excitation of 450 and 535nm, respectively. (E) Overlay staining of 8-oxodG (green) and PI (red) showed orange staining in the nucleus. (F) H&E staining of corresponding kidney sections from each tissue show kidney sections from all four groups of normal healthy subject, nodular glomerulosclerosis, glomerular hypertrophy and thickening tubular in diabetic patients, tumor with clear cell carcinoma and normal tubular cells (in adjacent tissue) in cancer patients and tumor with clear cell carcinoma with prominent cell borders and prominent vasculature in kidney section of cancer patients with diabetes. (G) Histogram showed percentage of total number of nucleus stained with 8-oxodG in kidney sections from all four groups. Significant difference from normal healthy kidney tissue to other groups is indicated by *P<0.01.

Deficiency in DNA repair enzyme OGG1 has important functional consequences, compromising the ability of cells to repair DNA. The steady-state levels of 8-oxodG are significantly higher in tissues of OGG1 knockout mice compared with wild-type animals. 8-OxodG is the major form of oxidative DNA damage and the ability of DNA adducts such as 8-oxodG to cause mutations is well documented. Our recent published data show that 8-oxodG was significantly accumulated in renal proximal tubular cells treated with high glucose and kidney cortex of diabetic rat [24]. In addition, these data show that decrease in OGG1 staining in kidney cortex of diabetic rats compared with control rats [24]. Significant decrease in OGG1 expression was detected in kidney tissue of diabetic patients compared to normal kidney tissues from healthy subjects. Kidney tissue from cancer patients with or without diabetes showed an extreme decrease in the protein expression of OGG1 compared to kidney tissue from diabetic patients (Fig. 4A&B). Data in Fig. 4C showed that 8-oxodG immunofluorescence staining was highly abundant in kidney section of cancer with diabetes (RCC+Diab) more than cancer without diabetes (RCC) or diabetes (Diab) alone compared to normal healthy subjects (Fig. 4C). We have observed that the majority of DNA damage is localized in tubular cells. H&E staining from the same kidney sections of all four groups was performed to show the changes in cell structure in diabetic kidney tissue and tumor histology of kidney cancer with or without diabetes (Fig. 4F). Percentage of total number of stained nucleus with 8-oxodG show increased in DNA damage in patients with kidney cancer with diabetes compare to patients with kidney cancer or diabetes alone (Fig. 4G).

## DISCUSSION

In this study, we provide evidence that hyperactivation of Akt/mTOR and significant decreased in tuberin resulted in significant decrease in DNA repair enzyme (OGG1) and accumulation of oxidative DNA damage in kidney cancer patients with diabetes. Hyperactivation of Akt resulted in significant decrease in tuberin and hyperactivation of mTOR suggesting that tuberin is a major tumor suppressor protein involved in the development of kidney cancer. Phospho-Akt/tuberin and total tuberin were mainly identified in tubular cells indicating the type of cell from which the cancer developed. In addition, our data showed that decrease in tuberin resulted in a significant increase in a cell proliferation marker, Ki67, in tubular cells of kidney cancer patients with diabetes suggesting that tuberin, a major protein, controls the cell proliferation. Our data also showed that significant decrease in OGG1 in kidney cancer patients with diabetes resulted in an accumulation and a significant amount of oxidative DNA damage, indicating the role of DNA repair enzyme in tumorigenesis. These findings indicate that Akt/tuberin/Ki67/mTOR/OGG1 pathway highly contributed in the tubular cells damage in diabetic kidney to initiate tumorigenesis.

In diabetes, the renal tubular cells are subject for both direct and indirect insults. Tubular thickening and interstitial lesions are prominent in diabetic patients [33]. Akt is activated by phospholipid binding and activation loop phosphorylation at Thr^308^ by PDK1 and by phosphorylation within the carboxyl terminus at Ser^473^. Phosphorylation of Akt at Ser^473^ has been identified as a mammalian target of rapamycin (mTOR) in a rapamycin-insensitive complex with rictor. We have shown, recently, that treated renal tubular cells with high glucose (25mM) and induced diabetes in rats promotes an increase in Akt phosphorylation and leads to phosphorylation of tuberin at Thr^1462^. High glucose activates the Akt/mTOR signaling cascade to stimulate protein synthesis [34,35]. Thus, activation of Akt represents a very proximal step in the intracellular signaling pathway triggered by high glucose. Phosphorylation of tuberin by Akt affects its function through at least two mechanisms: first, phosphorylation decreases the activity of tuberin; second, phosphorylation destabilizes tuberin by disrupting the complex formation between hamartin and tuberin resulting in the ubiquitination of free tuberin and its degradation by the proteosome [35]. The fact that the acute exposure of cells to high glucose is sufficient to elicit proliferation and then apoptosis of human proximal tubule epithelial cells. This suggests that episodes of increased glucose may contribute to cell injury and to epithelial cell dysfunction. On the other hand, our data showed that chronic exposure of tubular cells to high glucose in diabetic patients resulted in significant activation of Akt/mTOR pathway and inactivation of tuberin that lead to an increase in cell proliferation. In addition, decrease in tuberin resulted in an increase in cell proliferation. These proliferative cells express less DNA repair protein (OGG1) that lead to accumulate a significant amount of oxidative DNA damage 8-oxodG to be carried over to the new generated cells. Therefore, tuberin plays a major role in regulation DNA repair pathway linking high glucose to DNA damage. Collectively, the data indicated that long term chronic exposure of the kidney to high glucose in diabetic patients acts through Akt/tuberin/mTOR pathway to downregulate DNA repair OGG1, resulting in the accumulation of oxidized DNA, 8-OxodG. 8-OxodG is known to be a sensitive marker of oxidative DNA damage and of the total systemic oxidative stress in vivo [36]. Increased number of 8-oxodG–positive islet cells was found in the human pancreas from type 2 diabetic subjects [37].

In summary, our data provide strong evidence that hyperglycemia lead to hyper-phosphorylation of Akt, inactivation of tuberin, overexpression of Ki67 and hyperactivation of mTOR that resulted in significant decrease of DNA repair enzyme OGG1 and accumulate a significant amount of oxidative DNA damage. This signaling cascade Akt/tuberin/ki67/mTOR/OGG1 plays a major role in accumulation the DNA damage in renal tubular cells. Long-term exposure of tubular renal cells to hyperglycemia in diabetic patients resulted in significant decrease in DNA repair function and significantly increased the accumulation of oxidative DNA damage that initiates kidney tumorigenesis. Our data shed light on the molecular mechanisms implicated in these events. The present study provides additional rationale for maintaining tight control of plasma glucose to prevent the risk of the development of kidney cancer in diabetes.

## MATERIALS AND METHODS

### Kidney tissues

Human kidney tissues from de-identified cases of control (accidental death of healthy people), diabetes, and cancer with or without diabetes were obtained from the Tissue Bank for Development Disorders (University of Maryland, Baltimore, Maryland, USA) and San Antonio Cancer Institute Core, San Antonio, TX. Six kidney tissues and kidney paraffin sections from each group were used for Western blot and immunestaining. The study has been ethically approved by the Institutional Review Board of The University of Texas Health Science Center at San Antonio, TX.

### Protein extraction and immunoblot analysis

Kidney homogenates were prepared as described previously [32]. Protein concentrations were determined with the Bradford assay [38] using bovine serum albumin as a standard. Western blot analysis was performed as described previously [39]. Phospho-tuberin, tuberin, phospho-p70S6k, p70S6K, phospho-Akt, Akt and Ki67, antibodies were from Cell Signaling (Beverly, MA); GAPDH antibody was obtained from Santa Cruz Biotechnology. After extensive washing of membrane with Tris-buffered saline Tween-20 buffer, anti-rabbit IgG conjugated with horseradish peroxidase was added at a 1:5000 dilution and incubated for 1 hour at room temperature. An enhanced chemiluminescence kit (Amersham, NJ) was used to identify protein expression. Expression of each protein was quantified by densitometry using National Institutes of Health image 1.62 software and normalized to a loading control.

### Immunofluorescence staining

Phospho-Akt, phospho-p70S6K, phospho-tuberin, total tuberin and Ki67 expression was assessed by immunofluorescence as previously described [40]. Paraffin kidney sections (4 μm) were incubated with nonimmune donkey IgG to block nonspecific binding, then incubated with a primary antibody followed by FITC-labeled anti-rabbit IgG, followed by Alexa Fluor-labeled donkey anti-rabbit IgG (Chemicon, Temecula, CA). FITC green signals and Alexa Fluor red signals were detected using a filter with excitation at 535 nm and 594 nm, respectively. Controls consisted of PBS/BSA in place of primary antibody followed by detection procedures as outlined above. Kidney sections were viewed and photographed using an Olympus Research microscope equipped for epifluorescence with excitation and band pass filters.

### Immunofluorescence staining of 8-oxodG

The amount of 8-oxodG was determined by immunostaining in kidney sections from all four groups as described previously [41]. Acetone-fixed frozen kidney sections (4 μm) were incubated with non-immune donkey IgG to block non-specific binding then incubated with mouse antibody against 8-oxo-dG (1:200) conjugated with fluorescein isothiocyanate (FITC) (Biodesign International, Saco, ME). The slides were reacted with Vectashield Mounting Medium (Vector Laboratories, Burlingame, CA). FITC green signals for 8-oxodG were detected using a filter with excitation range 450–490 nm and propidium iodide (PI) red signals for nuclear DNA using a filter with excitation at 535 nm. Sections were viewed and photographed using an Nickon microscope equipped for epifluorescence with excitation and band pass filters optimal for FITC and PI. To demonstrate staining specificity, control kidney sections were stained without primary antibody.

### Histology of kidney

Formalin-fixed kidneys were embedded in paraffin 4 μm sections stained with haematoxylin and eosin for histological examination by light microscopy. All kidney cancer tissues were examined for clear cell type and diabetic tissues for glomerulus and tubular alterations.

### Statistics

Data are presented as mean ± standard error. Statistical differences were determined using ANOVA followed by Student Dunnett's (Exp. vs. Control) test using 1 trial analysis. *P-values less than 0.01 were considered statistically significant.
